# Home-Based Transcranial Direct Current Stimulation in Primary Progressive Aphasia: A Pilot Study

**DOI:** 10.3390/brainsci14040391

**Published:** 2024-04-17

**Authors:** Kyriaki Neophytou, Kelly Williamson, Olivia Herrmann, Alexandros Afthinos, Jessica Gallegos, Nadine Martin, Donna C. Tippett, Kyrana Tsapkini

**Affiliations:** 1 Department of Neurology, Johns Hopkins University School of Medicine, 600 N. Wolfe Street, Phipps 488, Baltimore, MD 21287, USA; kneophy1@jhu.edu (K.N.); kwill261@jhmi.edu (K.W.); oherrma1@jhu.edu (O.H.); afthinos.a@gmail.com (A.A.); jgallegos@jhmi.edu (J.G.); dtippet1@jhmi.edu (D.C.T.); 2 Cooper Medical School of Rowan University, Rowan University, 401 Broadway, Camden, NJ 08103, USA; 3 Department of Communication Sciences and Disorders, Temple University, 1701 N. 13th Street, Philadelphia, PA 19122, USA; nadine.martin@temple.edu; 4 Department of Physical Medicine and Rehabilitation, Johns Hopkins University School of Medicine, 600 N. Wolfe Street, Phipps 174, Baltimore, MD 21287, USA; 5 Department of Otolaryngology-Head and Neck Surgery, Johns Hopkins University School of Medicine, 601 N. Caroline Street, Baltimore, MD 21287, USA; 6 Department of Cognitive Science, Johns Hopkins University, 3400 N. Charles Street, Baltimore, MD 21218, USA

**Keywords:** primary progressive aphasia, verbal short-term memory, working memory, transcranial direct current stimulation, home-based tDCS

## Abstract

Background: This study aims to determine (a) if home-based anodal transcranial direct current stimulation (a-tDCS) delivered to the left supramarginal gyrus (SMG) coupled with verbal short-term memory/working memory (vSTM/WM) treatment (“RAM”, short for “Repeat After Me”) is more effective than sham-tDCS in improving vSTM/WM in patients with primary progressive aphasia (PPA), and (b) whether tDCS effects generalize to other language and cognitive abilities. Methods: Seven PPA participants received home-based a-tDCS and sham-tDCS coupled with RAM treatment in separate conditions in a double-blind design. The treatment task required participants to repeat word spans comprising semantically and phonologically unrelated words in the same and reverse order. The evaluation of treatment effects was carried out using the same tasks as in the treatment but with different items (near-transfer effects) and tasks that were not directly related to the treatment (far-transfer effects). Results: A-tDCS showed (a) a significant effect in improving vSTM abilities, measured by word span backward, and (b) a generalization of this effect to other language abilities, namely, spelling (both real words and pseudowords) and learning (retention and delayed recall). Conclusions: These preliminary results indicate that vSTM/WM intervention can improve performance in trained vSTM/WM tasks in patients with PPA, especially when augmented with home-based tDCS over the left SMG.

## 1. Introduction

Primary progressive aphasia (PPA) is a neurodegenerative condition characterized by worsening language deficits that negatively affect communication abilities, reflecting progressive atrophy of parts of the brain that are related to speech and language [1,2]. PPA can be classified into three main variants, semantic (svPPA), nonfluent agrammatic (nfaPPA), and logopenic (lvPPA) variants, which are frequently associated with different pathologies [1]. In this study, we included patients with nfaPPA and lvPPA, because our study targeted verbal short-term memory/working memory (vSTM/WM) deficits, which are prominent in both of these variants. LvPPA is characterized by impaired word retrieval on both confrontation naming and connected speech tasks and impaired phrase and sentence repetition, the latter of which is thought to reflect the underlying cardinal feature of this variant: impaired verbal phonological short-term memory [1,3]. Atrophy typically occurs in the posterior temporal cortex and left parietal lobule [1,4,5,6]. The underlying neuropathology in this variant is commonly Alzheimer’s disease (AD) pathology [7,8,9]. NfaPPA is primarily characterized by nonfluent, effortful, agrammatic speech and/or motor speech disorder [apraxia of speech (AOS)] [1,4,10], although poor vSTM can also be seen [11,12]. Atrophy is observed in the left inferior frontal gyrus (IFG), insula, and supplementary motor areas [1,4,7,13]. Tau-positive pathology is the underlying pathology in 70% of those with nfaPPA [14]. In contrast to the other two variants, vSTM performance remains relatively spared in svPPA, especially in the early stages of disease progression [11,12], and therefore, this variant was not included in this treatment study. 

Most prior interventional studies in PPA have focused mainly on the development of behavioral therapies to slow language decline, and numerous studies (mostly targeting verbal naming) have reported improved therapy outcomes [15]. Transcranial direct current stimulation (tDCS) has recently been used to augment language therapy in PPA with promising results, as we and others have shown [16,17] (for recent reviews and meta-analyses, see [18,19]). Beneficial effects of anodal tDCS on oral naming have been found by stimulating the left dorsolateral prefrontal cortex (DLPFC) combined with naming therapy in nfaPPA [16,20], stimulating the left temporoparietal cortex with concurrent individualized speech therapy in lvPPA, svPPA, and AD [21], and by stimulating the left inferior parietal lobe during picture naming training in all variants of PPA [22]. Roncero and colleagues compared anodal tDCS of the left DLPFC and anodal tDCS of the left inferior parietal lobe (IPL), along with picture naming training, and found improved spontaneous oral naming for both montages immediately post-treatment [23]. At two weeks post-treatment, spontaneous oral naming was superior following IPL compared with DLPFC stimulation. In addition, there was significant improvement for untrained items only after IPL stimulation. Gervits and colleagues employed tDCS stimulation of the left frontotemporal region to investigate the effect of tDCS on a wide range of language skills in lvPPA and nfaPPA [24]. They found significant benefits in picture naming that persisted for 12 weeks post-stimulation, as well as benefits in grammatical speech production, repetition, grammatical comprehension, and semantic processing. Promising findings regarding anodal tDCS stimulating the left IFG with written word naming/spelling treatment versus sham have been demonstrated as well [17,25,26,27,28,29,30]. More recently, Wang et al. showed generalization effects of tDCS to the left IFG paired with lexical/semantic retrieval intervention for oral and written naming and semantic fluency [31].

We and others have also identified several predictors that determine which patients with PPA will benefit most from tDCS. Several variables have been identified: (a) clinical variables such as the PPA variant (nfaPPA and lvPPA showed larger effect sizes) [30], (b) behavioral variables such as the initial performance in cognitive/language tasks, particularly vSTM and vWM [25], and (c) neuroimaging variables, such as before-treatment structural measures of brain volumes [26], white matter integrity [32], and functional connectivity [33]. In these studies, the most significant predictors of generalization of tDCS benefits to untrained items and tasks (those that are not targeted in therapy) were vSTM/WM performance, specifically verbal learning, as well as updating and monitoring (i.e., language and executive functions) [25]. Similarly, both the cortical volume and the white matter integrity of vSTM/WM neural substrates, specifically of the left supramarginal gyrus (SMG), predicted generalization of tDCS effects. Thus, findings from several investigations converge on the conclusion that generalization of tDCS benefits beyond treated tasks and stimuli in PPA depends to a great extent on vSTM/WM and its neural substrates (i.e., the left SMG). 

The importance of vSTM/WM in language processing has been highlighted in post-stroke aphasia [34]. vSTM/WM is responsible for the temporary storage and retrieval of verbal stimuli and is usually impaired in post-stroke aphasia [35]. Sometimes, vSTM is considered to be the component of vSTM/WM that is involved in maintenance but not manipulation of verbal stimuli, whereas WM is responsible for the mental manipulation of representations in vSTM (for discussion see [36,37]). In comparison to vSTM, WM is more closely related to specific aspects of attention and executive functioning such as updating, shifting, and inhibiting verbal information [35]. Notably, impairments in vSTM/WM have been associated with deficits in word and sentence comprehension and production [35], and individuals with post-stroke aphasia exhibit reduced vSTM capacity and WM abilities [34]. Similarly, recent studies have also shown that vSTM/WM and other executive functions are compromised in PPA, even though it is primarily considered a language disorder [38].

Interventions targeting vSTM/WM in persons with aphasia are recent developments [39], although the preliminary evidence demonstrates that these interventions show generalization to untreated tasks for some participants. Improvements in sentence comprehension have been observed in several studies using working memory tasks as treatment [35,40,41,42]. Using word and word-sequence repetition with response delay as a vSTM training task, Martin and colleagues reported improvements in naming for some participants, as well as sentence comprehension and discourse [34,43,44]. Martin and colleagues emphasize that the addition of memory load to the language tasks enables the direct involvement of processing aspects of language function (e.g., activating, maintaining, and retrieving the semantic or lexical representations of words), which are impaired to different extents across people with aphasia [43]. 

With regard to the neural substrates of vSTM/WM, the left SMG (Brodmann’s Area [BA] 40) is associated with the temporary storage of linguistic, mostly phonological [45,46], as well as graphemic [47] information. Diffusion tensor imaging (DTI) [48] and resting-state connectivity [49,50] have shown that the connections in the human cortex are consistent with the view that the left SMG connects via the third branch of the superior longitudinal fasciculus (SLF III) with pars opercularis (BA 44), and the adjacent most rostral inferior BA 6 (verbal premotor cortex) [48,51,52,53]. The left SMG has been considered an area involved in vSTM and particularly in the processing of phonological information [54]. It has been found to be involved in phonological aspects of key language tasks, such as repetition, naming, and spelling. As mentioned above, a key element to Martin and colleagues’ work targeting vSTM in post-stroke aphasia [34,43] is an increase in the vSTM “load” (i.e., adding response delays or increasing the number of items to be retained or monitored), which has also been found to involve inferior parietal areas, including the left SMG [54,55]. We thus hypothesized that stimulation of the left SMG with a-tDCS will improve vSTM abilities and induce generalization to some, untrained, language-specific tasks.

In the present pilot and proof-of-concept study, we tested, for the first time to our knowledge, this hypothesis in patients with PPA. In particular, the present study aimed to determine (a) whether a-tDCS of left SMG combined with vSTM/WM treatment will improve vSTM performance more than sham-tDCS in patients with nfaPPA and lvPPA, and (b) whether the treatment effects will generalize to other language functions. Of note, the COVID-19 pandemic prevented many patients and care partners from traveling for consecutive tDCS + behavioral therapy sessions, which ultimately put a halt to in-person treatment sessions. Additionally, there is little research documenting the efficacy of remotely supervised tDCS to improve specific speech, language, or cognitive outcomes. To date, previous research on tDCS has primarily focused on in-person treatments with high-definition or standard tDCS. Therefore, this is also the first study, to our knowledge, in which standard tDCS treatment for a neurodegenerative condition was administered remotely (home-based tDCS) under clinical supervision via video-calling, which allowed for greater convenience for both patients and their care partners. 

## 2. Materials and Methods

### 2.1. Participants 

Individuals were eligible to participate if they were native English speakers, had completed a high school education, did not have developmental disorders (e.g., dyslexia) or other neurological conditions (e.g., stroke), were right-handed, and had a formal criteria-based diagnosis of PPA of any severity [1]. Participants were referred by physicians from specialized PPA and frontotemporal dementia (FTD) centers across the United States. Referrals were generally based on neurological examination, cognitive–linguistic testing, and neuroimaging measures, including MRI and positron emission tomography (PET). The mean age of participants was 68.71 years ± 6.99, and the mean Clinical Dementia Rating Scale Sum of Boxes [56] (mean for controls age 69.1 ± 10.2 years = 0.04, range 0–0.5) was 4.35 ± 2.58 (see Table 1).

### 2.2. Design of Treatment Protocol

**Figure 1:**
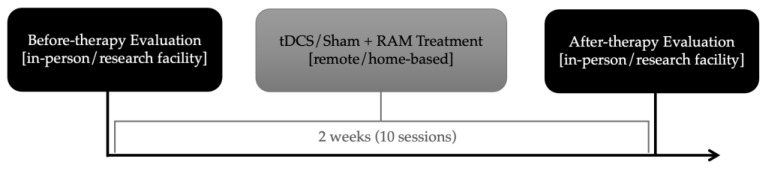
Timeline of the study protocol: before- and after-therapy evaluations performed in-person at the research facility, with 2 weeks (10 sessions) of tDCS/Sham stimulation paired with RAM (“Repeat After Me”) therapy in-between evaluations, performed remotely, from home.

In our double-blind randomized design protocol, participants received remote a-tDCS over the left SMG coupled with “Repeat After Me” (“RAM”) treatment. Randomization of stimulation condition took place before treatment (tDCS or sham condition). The randomization was carried out in groups of four participants, and within these groups, we used simple randomization, i.e., flipping of a coin. The executer was not aware of the block size that the allocator used to prevent guessing of the next participant’s condition. For this pilot study, there was no stratification by variant. Of the 7 participants, 4 were randomized to receive a-tDCS condition first, and 3 received sham-tDCS first, all paired with vSTM/WM behavioral treatment (RAM). Before- and after-treatment evaluations took place at the Johns Hopkins Hospital, while treatment sessions were performed at home for two weeks. The timeline of the study protocol is presented in Figure 1. The study was approved by the Johns Hopkins Medicine Institutional Review Board (IRB00201027), and all participants provided written consent.

### 2.3. tDCS

Active and sham remote tDCS were delivered using Soterix Medical mini-CT, which captures the features of the clinical trial standard 1 × 1-CT in a deployable platform but can be used remotely as a home-based tDCS device. The anode was placed over the left SMG (centering at CP3, respectively, according to the EEG 10–20 electrode position system), and the cathode was placed on the right cheek [57]. Current was delivered at 2 mA for 20 min for tDCS or 30 s ramp-up for sham, starting concurrently with treatment. After the ramping, in the sham condition, the current intensity was dropped to 0 mA, eliciting a transient (typically 30 s) tingling sensation, which is the same as what is reported in real tDCS [30,58].

Participants generally tolerated tDCS well, with the only reported side effects being an initial tingling or itching sensation for some participants [59], reported for both tDCS and sham conditions. When participants were debriefed at the conclusion of treatment, they were at chance in differentiating between conditions, based on the difference in sensation. The inability of the patients to differentiate between the two conditions suggests that blinding was successful. However, when caregivers were asked, they were 100% accurate based on differences in patient’s performance. Each participant received a set of daily changing unique five-digit codes (provided by the manufacturer of the tDCS stimulator), which instructed the stimulator to deliver either active-tDCS or sham-tDCS. To ensure double-masking (which, in fact, was triple-blinding), code assignments were not disclosed during the tDCS intervention to either patient, care partners, or experimenters [evaluators and treating speech–language pathologists, (SLPs)]. At study completion, the list was decoded to identify the participants in the active and sham groups. The tDCS stimulator did not display any information to identify the sham or active stimulation.

The administration of the home-based tDCS (both the equipment setup and the entire stimulation session) was monitored by a trained SLP via videocall for the entire duration of the session, for all sessions. The caregiver was responsible for preparing the tDCS headband after they received training (i.e., a live demonstration) when they visited our lab in-person for the before-therapy evaluation session. In addition to the live demonstration, they also received written instructions. At the beginning of each stimulation session and initial tDCS setup, the clinician would guide the caregiver through troubleshooting any problems that were encountered. To facilitate the process, the headband was also marked to ensure proper alignment. For the stimulation to start, the caregiver had to use one of the daily changing unique codes mentioned above, given to them by the SLP, for each treatment session. The caregiver was present throughout each session. 

### 2.4. Behavioral Treatment

#### 2.4.1. Treatment Outline and Stimuli

We used a novel intervention in PPA, “RAM”, which is based on a vSTM/WM intervention by Martin and colleagues in post-stroke aphasia [34,43]. The treatment consisted of 1 session each weekday for two weeks, for a cumulative total of 10 treatment sessions; each session lasted approximately 45–60 min. This training task required participants to repeat word spans of different lengths that comprised semantically and phonologically unrelated words, first in the same order and then in reverse order. The repetition of word spans in the same order targeted vSTM abilities, while the repetition of word spans in the reverse order targeted WM abilities, since, as discussed above, vSTM is considered to be involved in maintenance but not manipulation of verbal stimuli, whereas WM is responsible for mental manipulation of representations in vSTM. By eliminating repeated exposure to the same word list, the task trains the processes of activating and maintaining representations of words to achieve generalization rather than learning a specific list of words. 

Word lists for repetition were constructed using the same procedures as in Martin’s study, and the actual items were selected from the same list [43]. Each word span (e.g., 2-word spans, 3-word spans, etc.) was semantically and phonologically unrelated to the others, and only concrete nouns with a maximum of 3 syllables were used. There were 10 items for each word span length (e.g., 10 × 2-word spans, 10 × 3-word spans, etc.). The starting list length for the word span (typically pairs and triplets) was based on each participant’s initial performance in the Temple Assessment of Language and Short-Term Memory in Aphasia (TALSA) [34]. This protocol ensured individualized treatment so that participants were challenged yet could perform the task and show improvement. Virtual homework (via Microsoft PowerPoint) was provided after each session, modeling the treatment procedures.

#### 2.4.2. Treatment Procedures

Behavioral therapy was concurrent with active or sham stimulation. Treatment session was conducted as follows: (1) the SLP informed the participant which word span length was to be trained (e.g., pairs, triplets). The participant was presented with the first word span in a set and instructed to repeat it in the same order (i.e., forward) (e.g., for triplets, “dog-table-car”, the target response is “dog-table-car”). (2) The SLP recorded the word(s) that were recalled both in hard copy and digitally and provided verbal feedback. (3) If a participant repeated the word span correctly two consecutive times, the next span in the set was presented. If the word span was not correctly repeated two consecutive times, the SLP presented the same span again (up to five times), and the participant attempted to repeat it. The SLP reproduced the participant’s errors and modeled correct repetition. (4) If a participant reached 70% correct within a given set (e.g., 7/10 correct pairs), then the next level was introduced (e.g., triplets), and conversely, if 70% correct was not obtained, then the next level was not introduced. (5) Following completion of all word spans in the same order, the SLP informed the participant that they would transition to repeating word spans in the reverse order (e.g., if the SLP said “cat-window”, the participant would have to say “window-cat”). The same procedures were applied to reverse word span repetition as in the same-order word span repetition. 

### 2.5. Outcome Measures

Evaluations were conducted before and immediately after treatment. Our treatment evaluation was divided into two types of outcomes: primary outcomes and secondary outcomes. The primary outcomes assessed near-transfer effects, that is, effects that are directly related to the treatment. Our primary outcomes consisted of the same type of tasks as the treatment, but different items were used compared to the treatment (i.e., items on which participants were not trained). The secondary outcomes assessed far-transfer effects, that is, effects that are not directly related to the treatment (i.e., the effect of training on language performance beyond WM). More information about the different outcomes is provided below. Appendix A presents the performance of every individual in each of these tasks, while normative data (based on the literature) can be found in Appendix B.

#### 2.5.1. Primary Outcomes: Forward and backward Word Spans

The primary outcome measures assessed near-transfer effects using Subtest 14 (Word Repetition Span) from TALSA (Temple Assessment of Language and Short-Term Memory in Aphasia) [60]. Participants were tested on word span sets ranging from 2 words to 5 words, and the same items were administered both in the forward and the backward order (henceforth, TALSA Forward and TALSA Backward). A time delay of at least 30 min was implemented between the administration of TALSA Forward and TALSA Backward. In both cases, pass criteria for the word span set were 50% of the spans being correct in the same order. For example, if the participant repeated 5/10 sets correctly within the level of 3-word spans, they proceeded to the 4-word span level. Word span calculation included all spans that the participant attempted until they failed to reach a 50% score, as indicated in [34]. With this rule-based scoring system, 2 points were designated for each correct 2-word set, 3 points for each 3-word set, 4 points for each 4-word set, and 5 points for each 5-word set. For example, if at the evaluation a patient showed 60% accuracy with the 2-word sets (6/10 sets correct at the 2-word level) and 10% accuracy with 3-word sets (1/10 sets correct at the 3-word level), their score equaled 15 ((6 × 2 = 12 points) + (1 × 3 = 3 points)). The ceiling for the span lengths varied by participant due to varying disease severity and vSTM impairment.

#### 2.5.2. Secondary Outcomes: Generalization Tasks

The secondary outcome measures assessed far-transfer effects on a variety of tasks. Specifically, we assessed performance in spelling, learning, syntactic processing, sentence repetition, and object naming. 

Real-word spelling was assessed using a custom list of thirty items, divided into high phoneme–grapheme and low phoneme–grapheme probability words. Phoneme–grapheme probability describes the probability with which a given phoneme maps onto a grapheme (e.g., f for /f/ is a more probable mapping than ph for /f/), and it is, thus, used as an index of the spelling regularity of an item. Pseudoword spelling was assessed using twenty items from the JHU Dysgraphia Battery [61]. For each item (both real words and pseudowords), spelling accuracy was evaluated at the letter level using the Open Brain AI platform [62]. Specifically, every letter in the target response received a score based on the patients’ response, and the average score across all letters provided the percentage accuracy scores per item [63,64,65]. The item score captured the orthographic (for real words) and phonological (for pseudowords) distance between the patient’s response to the target response. For more information on the spelling scoring process for real words and pseudowords, see [66]. Item scores were then averaged across the relevant items to get an overall spelling accuracy score. Four different average scores were calculated: (i) real words—all items, (ii) real words—high-probability items, (iii) real words—low-probability items, and (iv) pseudowords. 

Learning was assessed using the Rey Auditory Verbal Learning Test (RAVLT) [67]. Two different scores were calculated: (i) retention, calculated by subtracting the raw score (i.e., the number of items recalled) of Trial 5 from that of Trial 8 of RAVLT, and (ii) delayed recall, which was the raw score at Trial 8. Syntactic processing was assessed using the SOAP (Subject, Object, Active, Passive) test [68], and syntactic processing was evaluated using Subtest 7 (Sentence Repetition) from the TALSA. For both of them, raw scores were used. Finally, naming was assessed using the Boston Naming Test (BNT) [69]. Scoring for naming was calculated using the percentage accuracy scores per item based on the phonological distance between the patient’s response to the target response, using the Open Brain AI platform [62]. Item scores were then averaged together to get an overall spelling accuracy score.

### 2.6. Statistical Analysis

For each outcome measure, three one-tailed t-tests were performed: (i) before vs. after treatment for the a-tDCS group, (ii) before vs. after treatment for the sham-tDCS group, and (iii) period difference (after minus before) in a-tDCS vs. period difference in sham-tDCS. The statistical significance threshold was set at *a* = 0.05. Tests (i) and (ii) evaluated the hypothesis that performance is better after treatment compared to before, for the a-tDCS and the sham-tDCS groups, respectively. Test (iii) evaluated the hypothesis that a greater change would be seen after treatment for the a-tDCS group compared to the sham-tDCS group. The data analyzed in this pilot study were preliminary data, which were subsequently submitted with a grant proposal. 

## 3. Results 

Table 2 presents the results for all *t*-tests (for effect sizes, see Appendix C). For the primary outcomes, a-tDCS showed a statistically significant effect (*p* = 0.041) for TALSA Backward. For the secondary outcomes, a-tDCS showed a statistically significant effect for all four spelling measurements (all real words: *p* = 0.005; high-probability real words: *p* = 0.045; low-probability real words: *p* = 0.003; pseudowords: *p* = 0.029), while the sham-tDCS group did not show any significant effects. The a-tDCS group showed a greater effect compared to the sham-tDCS effect for all four spelling measurements (all real words: *p* = 0.002; high-probability real words: *p* = 0.058; low-probability real words: *p* = 0.001; pseudowords: *p* = 0.102). The a-tDCS group also showed a greater effect compared to the sham-tDCS effect for the two learning measurements (Retention: *p* = 0.068; Delayed Recall: *p* = 0.075).

## 4. Discussion 

This is the first, to our knowledge, proof-of-concept study that aims to determine the feasibility and potential benefit of home-based tDCS in PPA. Home-based tDCS provides an important treatment option in a condition without disease-modifying treatments and limited symptomatic treatment options (limited speech therapy and in-person neuromodulation). We and others have previously shown that in-person tDCS in PPA augments behavioral treatment outcomes, especially for oral and written naming (for relevant reviews and metanalyses, see [18,58,70]). However, it was not known whether this approach can be carried out remotely from home, minimizing insurance costs and care partners’ burden. These first, preliminary results show that (1) home-based tDCS application is feasible in patients with PPA; (2) home-based a-tDCS can be more efficacious than sham-tDCS in improving performance in vSTM/WM after 10 sessions (i.e., 2 weeks) of tDCS + vSTM/WM treatment; (3) home-based a-tDCS can be more efficacious than sham-tDCS in the generalization of improvement in untrained abilities such as spelling. These results primarily highlight the feasibility and also the potential effectiveness of remote, home-based tDCS in the treatment of PPA.

The present effects of a-tDCS aligned with prior evidence that working memory treatment using repetition as the treatment task can lead to a generalization of treatment effects [35,42,43]. All language tasks engage STM processes to some extent to maintain activation of word representations of the course of that task. Martin et al. reported that naming accuracy improved and nonword productions decreased on picture naming with response delays, showing that it is effective to add memory load or time delays to tasks other than repetition [71]. Repetition tasks, which are used in many vSTM studies including this study, are a good choice for improving comprehension and discourse abilities, as repetition engages both input and output processes. 

vSTM abilities are engaged to different extents across tasks. In the context of Dell’s interactive activation model [72], input processes that initiate repetition engage top-down processes as feedback from the semantic to the lexical levels of representation in the later stages of repetition [73] (for an account of processes that support word repetition, see [74]). However, word repetition can proceed to a degree without access to or support from semantic representations, such as in transcortical sensory aphasia [75]. Thus, improvement in naming based on repetition training tasks would not be expected to be robust, depending on the strength of processing connections between input and output lexical–semantic pathways. In an effort to improve naming ability using a vSTM training approach, Martin and colleagues added the vSTM components (memory load) to a naming treatment task and observed robust improvements in naming for some participants, specifically those who showed a significant decline in repetition accuracy after a response delay before treatment [71]. These interventions, which have investigated the effects of adding vSTM to language tasks, have provided valuable information about aspects to consider (e.g., the nature of the ability to be improved and task used to stimulate processing) when developing a treatment protocol. Similarly, future studies may determine that modifications of vSTM training tasks will be needed for different variants of PPA. 

In our study, the participants did not demonstrate improvement in object naming (BNT task), perhaps because the treatment task does not engage lexical–semantic pathways as much as other tasks do. Other factors that might have played a role include the neurodegenerative nature of their disease or a difference in therapy frequency (i.e., Martin and colleagues treated participants with four sessions per week over three weeks or two sessions per week over six weeks, as opposed to five sessions per week for two weeks in our protocol). The implementation of a vSTM treatment should determine what language task, as the vehicle for the vSTM processing stimulation, would be the most effective in successfully improving the desired language ability. Ultimately, this could involve several tasks, each adapted to engage vSTM processes that support successful language comprehension and production.

Notably, our participants improved significantly in all measurements from the spelling to dictation task during a-tDCS, and the a-tDCS group also showed greater effects compared to the sham-tDCS group. High-probability words are considered to be regular, as they can be spelled by following the phoneme-to-grapheme conversion (PGC) rules (e.g., work), and low-probability words are considered to be irregular, because they do not follow the PGC rules but have rather arbitrary spellings (e.g., aisle). Previous neuroimaging and lesion studies place the neural substrates of the PGC processes (also called *orthographic working memory* or *orthographic buffer*) in the left SMG [47,76]. The results of this study showed that targeting vSTM through behavioral treatment in combination with stimulation of the left SMG can help the activation and maintenance of regular phoneme-to-grapheme mappings, a process that supports spelling across all items, both real words and pseudowords. On the other hand, the sham-tDCS group, which did not receive stimulation in the area that supports PGC, did not show a significant effect for spelling. In sum, the results of the current study, albeit preliminary, extent the findings reported by Martin et al. [71], who showed improvement in oral language production to the domain of written language production, which has been shown to be a rather sensitive measure of improvement in PPA [30].

Finally, marginally significant differences were observed for the a-tDCS group compared to the sham-tDCS group for our learning measurements (RAVLT Retention and RAVLT Delayed Recall), with the a-tDCS group showing a better learning performance compared to sham-tDCS. STM is an indispensable component of learning [77,78] and, as discussed above, the SMG has long been implicated in STM. The results reported here suggest that an improvement in STM abilities, driven by the stimulation of the left SMG, can have beneficial effects for learning as well. 

### 4.1. Specificity of Stimulation

PPA is a multifaceted disorder, with different variants, clinical characteristics, and patterns of atrophy. Although significant effort and resources have been devoted to the search for pharmacological treatments, there are no disease-modifying treatments to date [79,80]. Recent behavioral and neuromodulation treatment approaches, however, are improved and more specific: (a) they concentrate on a single cognitive process (i.e., vSTM/WM) and (b) target synaptic transmission. The proposed research is responsive to these by targeting vSTM/WM and employing tDCS with a network approach for evaluating brain targets, based on our current knowledge of brain network organization, in which neurodegenerative processes affect large-scale brain networks [81]. Many studies on tDCS have identified the principle of functional targeting [82], which emphasizes that active cells and networks are changed by tDCS (i.e., those activated by the concurrent behavioral task). This suggests that the efficacy of tDCS is based on the relationship between the concurrent behavioral task and the function of the area of stimulation and its connectivity. It seems that even stimulating an area that may be compromised, e.g., atrophied, there are still active cells in these areas, as well as in structurally or functionally connected areas, which will be affected by the stimulation if engaged in the targeted cognitive function by the concurrent treatment task [30,83]. Therefore, our hypothesis that tDCS over the left SMG, as part of the perisylvian language network and neural substrate of vSTM/WM, would improve performance in vSTM/WM tasks was confirmed, as TALSA backward showed improvement in the a-tDCS group only and not in the sham-tDCS group, and, for spelling, the a-tDCS group showed greater effects compared to the sham-tDCS group. This approach, in which stimulation targeting the affected area in combination with treatment targeting the affected cognitive function, aligns with previous studies showing that anodal tDCS over the left IFG improves naming [16], spelling [17], and syntax [19].

### 4.2. Feasibility of Remote Home-Based tDCS

The results of this preliminary and pilot study first and most importantly demonstrate the feasibility of using remote home-based tDCS in patient rehabilitation. The current findings also demonstrate the potential benefit of a treatment targeting vSTM/WM to improve these abilities in PPA patients and, second, the value in testing the efficacy of remote tDCS and RAM treatment on PPA patients in a larger-scale study. Across all seven participants, there were no reported adverse effects from tDCS, as has been consistently reported in the literature.

Additionally, the remote mini-CT devices captured the features of the clinical trial standard 1x1-CT in a deployable platform, with easy administration. All participants implemented treatment in their home setting and tolerated treatment well, with the only reported side effects being initial tingling or itching for some participants. This home-based tDCS treatment may also be advantageous over other non-invasive neuromodulation techniques, such as Transcranial Magnetic Stimulation (TMS). TMS has shown positive potential in PPA treatment, especially when the stimulation target is personalized [84], yet TMS administration requires hospital-based administration, and personalization of stimulation targets involves MRI image acquisition. Both of these elements make the administration of personalized TMS stimulation more challenging compared to home-based tDCS stimulation. Taking all this information into account, remote tDCS may increase the accessibility of treatment for those who are unable to travel due to financial, physical, or other personal limitations.

### 4.3. Limitations

There are several limitations to the present study. The most important limitation is the small sample size, which makes our results prone to individual effects that may not generalize in larger samples. Therefore, large-scale studies are needed, and now, an international multisite tDCS treatment study is underway (NCT05386394). Another important limitation is that given the small sample size, we were not able to investigate any variant differences that may exist, given the different atrophy distributions amongst variants. Nevertheless, this is the first study on PPA, and the purpose of this report is to show the feasibility and provide a proof-of-concept for home-based tDCS in a rare neurodegenerative disorder affecting language.

Another possible limitation of this study is that we did not examine neurophysiological changes due to tDCS or sham and how they correlate with atrophy in this small cohort. If one of the tDCS neurophysiological outcomes is changes in functional connectivity [28,85,86], and functional connectivity is not correlated to atrophy as has been shown by our group and others [87,88], then the different atrophy patterns may not affect the efficacy of tDCS. However, this is an empirical question that future research should address.

## 5. Conclusions

This study showed that remote tDCS, when used in conjunction with vSTM/WM treatment, improves vSTM/WM, as well as spelling and learning, in PPA. The results of this study provide additional evidence for the use of remote tDCS alongside language treatment to target STM/WM deficits in PPA. The present study provides the first proof-of-concept of the feasibility and efficacy of remotely supervised home-based tDCS in a rare neurodegenerative disorder. It also provides a basis for the larger-scale implementation of home-based tDCS paired with behavioral treatment. Further research with a larger sample is warranted to determine the effects of tDCS and vSTM treatment on patients with PPA.

## Figures and Tables

**Table 1:** Participant demographics.

Participant Code	Diagnosis	Age (years)	Sex	Treatment Group	Clinical Dementia Rating Scale
JSH	lvPPA	60	F	tDCS	9
ESS	nfaPPA	67	F	tDCS	3
RBL	lvPPA	77	M	tDCS	2.5
ANR	nfaPPA	62	M	sham	3
KST	lvPPA	73	F	tDCS	7
DWR	nfaPPA	77	M	sham	3.5
EHY	lvPPA	65	F	sham	2.5

**Table 2:** *P*-values with Degrees of Freedom and *t*-test statistic from relevant *t*-tests in parenthesis (*: *p*-value < 0.05; ~: *p*-value < 0.10) for each outcome of interest.

	a-tDCS + vSTM (*n* = 4)	sham-tDCS + vSTM (*n* = 3)	Difference
			a-tDCS vs. sham-tDCS
Word Span Forward (TALSA)	0.685 (t_3_ = −0.53)	0.262 (t_2_ = 0.77)	0.819 (t_5_ = −1.00)
Word Span Backward (TALSA)	0.041 * (t_3_ = 3.29)	0.181 (t_2_ = 1.17)	0.152 (t_4_ = 1.18)
Spelling Real Word—All	0.005 * (t_3_ = 5.98)	0.991 (t_1_ = −35)	0.002 * (t_4_ = 5.92)
Spelling Real Word—High-Probability	0.045 * (t_3_ = 2.47)	0.750 (t_1_ = −1.00)	0.058 ~ (t_4_ = 2.00)
Spelling Real Word—Low-Probability	0.003 * (t_3_ = 6.75)	0.946 (t_1_ = −5.80)	0.001 * (t_4_ = 6.94)
Spelling Pseudoword	0.029 * (t_3_ = 3.00)	0.539 (t_1_ = −0.12)	0.102 ~ (t_4_ = 1.52)
Retention (RAVLT)	0.252 (t_3_ = 0.76)	0.851 (t_2_ = −1.39)	0.068 ~ (t_5_ = 1.78)
Delayed recall (RAVLT)	0.303 (t_3_ = 0.57)	0.858 (t_2_ = −1.45)	0.075 ~ (t_5_ = 1.70)
Syntax (SOAP)	0.163 (t_3_ = 1.17)	0.346 (t_2_ = 0.46)	0.448 (t_5_ = 0.14)
Sentence Repetition (TALSA)	0.274 (t_3_ = 0.68)	0.761 (t_2_ = −0.87)	0.166 (t_5_ = 1.07)
Object Naming (BNT)	0.604 (t_3_ = −0.29)	0.334 (t_2_ = 0.50)	0.706 (t_5_ = −0.58)

## Data Availability

The original contributions presented in this study are included in Appendix A, and further inquiries can be directed to the corresponding author.

## Appendix A

Individual performance in each task, both for the primary outcomes (TALSA Forward and TALSA Backward) and the secondary outcomes (all other tasks). Performance is reported separately for “before” and “after” treatment. NaN = non-available data.

	**TALSA** **Forward**		**TALSA** **Backward**		**Spelling** **(All Real Words)**		**Spelling** **(LP)**		**Spelling** **(HP)**		**Spelling (Pseudoword)**	
	Before	After	Before	After	Before	After	Before	After	Before	After	Before	After
JSH	21	24	NaN	NaN	0.92	0.96	0.86	0.92	0.98	1.00	0.73	0.85
ESS	18	18	14	24	0.95	0.97	0.93	0.98	0.97	0.97	0.62	0.63
RBL	45	39	14	24	0.77	0.82	0.63	0.67	0.92	0.97	0.79	0.86
KST	46	45	28	31	0.89	0.94	0.82	0.90	0.95	0.98	0.80	0.89
ANR	72	74	29	29	0.98	0.96	0.96	0.93	1.00	1.00	0.84	0.89
DWR	33	30	17	26	NaN	NaN	NaN	NaN	NaN	NaN	NaN	NaN
EHY	20	29	22	23	0.99	0.97	0.97	0.95	1.00	0.99	0.82	0.75
	**RAVLT** **Retention**		**RAVLT** **Delayed Recall**		**SOAP**		**TALSA** **Sentence** **Repetition**		**BNT**			
	before	after	before	after	before	after	before	after	before	after		
JSH	−1	4	5	10	25	28	41	43	97.5	95.6		
ESS	−1	1	11	14	33	31	16	19	85.2	80.4		
RBL	−2	−1	2	4	28	29	34	34	35.7	39.4		
KST	−1	−4	12	7	28	33	48	46	88.4	89.3		
ANR	−1	−4	9	6	27	29	49	48	89.4	93.3		
DWR	−1	−2	7	6	24	30	45	42	96.7	93.2		
EHY	3	−14	15	0	32	28	35	36	93.6	96.7		

## Appendix B

Normative data for our outcome measures from the literature: ^a^ [34], ^b^ [68], ^c^ [89], ^d^ [90].

**Test**	**Normative Data** **(Age and Education in Years)**
TALSA Word Span Forward	Mean = 5.60 (SD 1.18), range = 3.00–7.00, median = 5.60, age mean = 56.33 (SD = 15.33), education range = 11 to 16 ^a^
Syntax (SOAP)	90–100% correct for unimpaired controls ^b^
Delayed recall (RAVLT)	Mean = 7.1 (SD 3.8), men ages 60–69 ^c^ Mean = 5.6 (SD 2.6), men ages 70+ Mean = 10.3 (2.3), women ages 60–60 Mean = 8.3 (2.1), women ages 70+
Retention (RAVLT)	Mean = 7.2 (SD 2.8), men ages 60–69 ^c^ Mean = 6.4 (SD 1.7), men ages 70+ Mean = 9.8 (1.6), women ages 60–60 Mean = 7.8 (1.8), women ages 70+
Object Naming (BNT)	Mean = 27.13 (SD = 2.06), age mean = 72.93, range 61–84, education mean = 13.73 ^d^
TALSA Sentence Repetition (unpadded)	Mean proportion correct = 1.00 (SD 0.01), range = 0.96–1.00, median = 1.00, age mean = 56.33 (SD = 15.33), education range = 11 to 16 years ^a^

## Appendix C

Effect sizes for all the *t*-tests conducted in the current study. The table shows Hedges’ g effect sizes, which is a corrected version of Cohen’s d. The correction is relevant for small sample sizes to reduce bias in the estimation of the population effect size. Values around and below 0.2 are considered small effects, values around 0.5 are considered medium effects, and values above 0.8 are considered large effects.

	**a-tDCS + vSTM (*n* = 4)**	**sham-tDCS + vSTM (*n* = 3)**	**Difference**
			**a-tDCS vs. Sham-tDCS**
Word Span Forward (TALSA)	−0.063	0.081	−0.645
Word Span Backward (TALSA)	0.960	0.560	0.769
Spelling Real Word (combined)	0.467	−4.000	4.104
Spelling Real Word—High-Probability	1.030	−0.571	1.382
Spelling Real Word—Low-Probability	0.348	−1.683	4.805
Spelling Pseudoword	0.599	−0.060	1.052
Retention (RAVLT)	0.452	−1.159	1.143
Delayed recall (RAVLT)	0.239	−1.323	1.091
Syntax (SOAP)	0.539	0.362	0.089
Sentence Repetition (TALSA)	0.050	−0.121	0.691
Object Naming (BNT)	−0.017	0.316	−0.373
